# Long‐Term Cardiovascular Impact of Postpartum Treatment After Hypertensive Disorders of Pregnancy: Population‐Based Cohort Study

**DOI:** 10.1111/1471-0528.70251

**Published:** 2026-04-22

**Authors:** Emmanuel Simon, Solène Tapia, Sonia Bechraoui‐Quantin, Jonathan Cottenet, Basky Thilaganathan, Catherine Quantin

**Affiliations:** ^1^ Université Bourgogne Europe, CHU Dijon Bourgogne, Service d'obstétrique et de gynécologie Dijon France; ^2^ Université Bourgogne Europe, Pathophysiology & Epidemiology of Cerebro‐Cardiovascular Diseases (PEC2), EA7460 Dijon France; ^3^ Université Bourgogne Europe, CHU Dijon Bourgogne, Service de Biostatistiques et d’Information Médicale (DIM) Dijon France; ^4^ Fetal Medicine Unit St George's University Hospitals NHS Foundation Trust London UK; ^5^ Université Bourgogne Europe, CHU Dijon Bourgogne, INSERM, CIC 1432, Module Épidémiologie Clinique Clinical Investigation Center, Clinical Epidemiology/Clinical Trials Unit Dijon France; ^6^ UVSQ, Univ. Paris‐Sud, Inserm, High‐Dimensional Biostatistics for Drug Safety and Genomics, CESP Université Paris‐Saclay Villejuif France

**Keywords:** antihypertensive therapy, cardiovascular complications, chronic hypertension, hypertensive disorders of pregnancy, pregnancy

## Abstract

**Objective:**

To assess whether early antihypertensive treatment after Hypertensive Disorders of Pregnancy (HDP) influences subsequent development of cardiovascular complications.

**Design and Setting:**

Population‐based nationwide cohort of health data set in France.

**Population:**

108 906 women with HDP (excluding pre‐existing Chronic Hypertension (CH)) who delivered between 2010 and 2014, with 35 878 (33%) receiving at least one antihypertensive treatment in the month after giving birth.

**Methods:**

Traditional Cox model, estimated 10‐year cardiovascular risk. Extended Cox Step Function model and Restricted Mean Survival Time evaluated time trends.

**Main Outcome Measures:**

New‐onset CH, heart failure, coronary, cerebrovascular, peripheral artery diseases and 2 composite events (one including CH, the other excluding it) over 10 years following giving birth.

**Results:**

Women receiving early postnatal antihypertensive treatment had a higher long‐term risk of complications over 10 years than non‐treated women (CH: aHR = 3.067, 95% CI [2.996–3.139]; composite event including CH: aHR = 3.025 [2.956–3.096]; composite event excluding CH: aHR = 1.451 [1.305–1.614]). Treated women had events earlier than non‐treated women, presenting a higher risk at the beginning of the postpartum period. The 10‐year absolute risk for CH remained high in both groups: 44% for treated women and 18% for non‐treated women.

**Conclusion:**

Our study shows that women receiving early postpartum antihypertensive treatment are at higher long‐term cardiovascular risk, with 44% of them having CH within 10 years. Besides, approximately 1 in 5 women non‐treated in the postpartum period subsequently developed CH, demonstrating that many high‐risk women are not being identified in the peripartum period and may be missing opportunities for timely intervention.

## 1 Introduction

Hypertensive disorders of pregnancy (HDP) are well‐established risk factors for future cardiovascular and cerebrovascular disease [1, 2, 3, 4]. Accumulating evidence indicates that the first year postpartum represents a particularly high‐risk period, during which women who had HDP are significantly more likely to develop chronic hypertension, heart failure, coronary heart disease, cerebrovascular disease and peripheral artery disease [5, 6]. Among these postpartum complications, the development of chronic hypertension is of particular concern, as it serves as a major modifiable risk factor for more severe long‐term events. Preventing or delaying the onset of chronic hypertension may offer a critical opportunity to mitigate the trajectory toward adverse cardiovascular and neurovascular outcomes. Early postpartum blood pressure control is therefore emerging as a key target for intervention [7]. Recent randomised controlled trials, including the SNAP‐HT and POP‐HT studies, have demonstrated the clinical benefits of enhanced self‐monitoring of blood pressure initiated shortly after delivery and sustained for up to 3 months postpartum [8, 9, 10, 11, 12, 13, 14]. These interventions were associated not only with improved mean blood pressure 3 months after stopping antihypertensive therapy, but also with favourable changes in cardiovascular imaging parameters, such as left ventricular mass and cardiac function [15].

Despite the promising clinical efficacy from prospective research trials, important questions remain regarding the clinical effectiveness of postpartum antihypertensive treatment in real‐world settings. In particular, the practical implementation and long‐term impact of early pharmacologic intervention remain poorly characterised. The aim of the current study was to compare the incidence of chronic hypertension and early cardiovascular complications among women who initiated antihypertensive treatment postpartum versus those who did not receive pharmacologic therapy. The analysis aimed to establish whether early postpartum antihypertensive treatment is associated with differences in subsequent cardiovascular outcomes.

## 2 Material and Methods

### 2.1 Database

This population‐based nationwide study was conducted using the French National System of Health Data (SNDS, Système National des Données de Santé) database, which contains individual, extensive and linkable, but anonymous data on healthcare use. It aggregates data on reimbursement for out‐of‐hospital care (treatments delivered), codes for Long‐Term Illnesses (LTIs) that entitle patients to coverage without coinsurance under the French national health insurance program, as well as data from the French national Programme de Médicalisation des Systèmes d'Information (PMSI) containing anonymous information covering both medical and administrative data from all hospitalisations in France. A unique anonymous identification number for each patient makes it possible to link all hospitalisations of the same person, allowing for longitudinal studies. The use of the PMSI database for the allocation of hospital budgets enhances data quality in terms of coherence, accuracy and exhaustiveness, due to internal and external validation of its quality [5, 16, 17, 18, 19]. Furthermore, more than 99% of births are recorded (excluding 0.4% of deliveries which occur out‐of‐hospital), which provides us with a near‐complete national capture of births with longitudinal linkage, allowing robust follow‐up.

Diagnoses identified during the hospital stay are coded according to the 10th edition of the International Classification of Diseases (ICD‐10), and surgical procedures are coded according to the French Common Classification of Medical Procedures (CCMP).

### 2.2 Ethics Approval

Written consent was not needed for this study because this was a retrospective study and the national data used were anonymous.

### 2.3 Population

The study included all women who gave birth (ICD‐10 code Z37) between April 1, 2010 and December 31, 2014 and who were diagnosed with HDP. To identify them, we searched for a diagnosis of preeclampsia (ICD‐10 code O14‐15) or gestational hypertension (ICD‐10 code O13) during the whole pregnancy. Only the first pregnancy with HDP of the period was retained in order to count each woman only once. Women with pre‐existing chronic hypertension were excluded. To do that, we excluded women who received antihypertensive treatment in the 6 months preceding pregnancy or during the 19 first weeks of pregnancy, or who were hospitalised for chronic hypertension during pregnancy (ICD‐10 codes O10‐11, I10‐13, I15). We also excluded women aged < 12 and > 57 years to retain all women who actually bore children, and women who died during the delivery stay (Figure S1).

### 2.4 Exposure

We identified all antihypertensive treatments dispensed in the 31 days (1 month) following maternity postnatal discharge for each woman (Table S2). We thus divided the women included into 2 groups: ‘treated women’ and ‘non‐treated women’. Treated women were those who received at least one antihypertensive treatment for at least the first month following maternity discharge, and non‐treated women were those who did not receive any antihypertensive treatment in the month following maternity discharge.

### 2.5 Outcomes

The main outcome was chronic hypertension. We identified it by three consecutive courses of antihypertensive treatment [20] (taking the date of the 3rd course of drug delivery as the time of diagnosis) or by hospitalisations coded with ICD‐10 codes I10‐I15. Other cardiovascular and cerebrovascular outcomes were searched for: heart failure; coronary heart disease, including angina pectoris, myocardial infarction, coronary revascularisation; cerebrovascular disease, including haemorrhagic and ischaemic stroke, transient ischaemic attack, carotid revascularisation; peripheral artery disease. All these cardiovascular and cerebrovascular events were identified from hospitalisations (Table S2), and tracked over 10 years from maternity discharge. If a woman was hospitalised for the same condition more than once during follow‐up, only the first event was considered. We also considered two composite events: the first included all cardiovascular and cerebrovascular events, including chronic hypertension; the second included all cardiovascular and cerebrovascular events, excluding chronic hypertension. These two composite events were defined as the first occurrence of at least one of the aforementioned conditions.

### 2.6 Variables

The following variables were identified for each woman: mother's age at delivery, gestational diabetes, morbid obesity (defined as BMI ≥ 40 kg/m^2^), smoking, severe alcoholism, family or personal history of cardiovascular diseases, and whether it was a single or multiple pregnancy. These variables were identified from all hospitalisations during pregnancy according to the ICD‐10 codes presented in Table S2. To identify smoking, we also looked for the use of nicotine substitutes during pregnancy or in the year preceding it. The presence of pre‐existing diabetes during pregnancy and in the year preceding pregnancy was assessed. To do that, we searched for hospitalisations, LTIs and delivery of antidiabetic drugs. We also looked for the presence of dyslipidaemia in the year preceding pregnancy, based on hospitalisations or delivery of related medication. The different drugs and LTIs considered are presented in Table S2. Finally, we looked for the French deprivation index developed by Grégoire Rey [21], used to measure the social environment in France.

### 2.7 Statistical Analysis

The characteristics and rates of outcomes over the entire study period were examined according to whether or not the women were treated with antihypertensive drugs during the month following maternity discharge. Continuous variables were presented as means±standard deviation (SD) and as medians and interquartile range, and categorical variables as percentages. The differences in baseline characteristics were tested using *χ* ^2^ tests for categorical variables and appropriate parametric or non‐parametric tests for continuous variables. We also calculated the Standardised Mean Differences (SMD). We looked at both Kaplan–Meier curves and number‐at‐risk tables. We assessed the risks of cardiovascular and cerebrovascular events over the 10 years in treated women compared with non‐treated women (reference), using standard Cox proportional hazards regression models to calculate hazard ratios (HRs) and 95% confidence intervals (CIs). The study participants who died during follow‐up were censored if the death was not related to one of the cardiovascular events. As the traditional Cox model is subject to conditions of validity, notably the assumption of proportional hazards, we have supplemented this analysis with two different and complementary modelling approaches. First, to account for the non‐proportionality of risks, we used the extended Cox regression with a step function model which allowed us to estimate the effect of the exposure variable in different time intervals. These intervals were chosen for their clinical significance: until 3 months, between 3 and 6 months, between 6 months and 1 year and annually thereafter. Second, we used the Restricted Mean Survival Time [22] (RMST) modelling which remains valid even when the proportional hazards assumption is not apparent. RMST is defined as the mean survival time up to a prespecified time, and to compare the effect of the treatment, the difference of RMST is used (rmstD) and can be expressed as the number of life years gained with the treatment (life expectancy difference). RMST analysis allowed us to take into account the cumulative information over 10 years. All our models were adjusted for maternal age, multiple pregnancy, gestational diabetes, morbid obesity, severe alcoholism, personal and family history of CVD, smoking, pre‐existing diabetes and dyslipidaemia.

We performed an additional analysis in which we calculated a propensity score based on the assessment of preterm HDP (gestational age < 37), severe HDP (identified by ICD‐10 codes O15, O14.2, N17, J81 between the diagnosis of HDP and delivery) and use of prenatal antihypertensive treatment (treatment delivered from the 20th week of pregnancy until delivery). The selection of the criteria for defining severe HDP was based on recommendations from the SFAR/CNGOF 2020 (France) [23], NICE 2023 (UK) [24] and ACOG 2020 (US) [25]. The three variables were then included in a logistic regression to calculate a propensity score for each woman. Each treated woman was then matched with a non‐treated woman with the closest score. We then performed the traditional Cox model on this matched cohort, unadjusted and adjusted for the associated comorbidities listed above (age, gestational diabetes, morbid obesity, severe alcoholism, personal or family history of CVD, smoking, pre‐existing diabetes and dyslipidaemia).

Several sensitivity analyses were performed to assess the robustness of our results. In the first one, we included women aged 15 to 49, in order to include only women of reproductive age according to current international operational definitions [26]. We also performed a sensitivity analysis including only women for whom we had access to the deprivation index. Finally, we performed a sensitivity analysis in which we excluded women with preterm or severe HDP in order to better assess the long‐term effect of antihypertensive medications. Two‐sided *p* values of < 0.05 were considered statistically significant. All analyses were done using SAS version 9.4 (SAS Institute, Cary, NC, USA).

## 3 Results

We identified 108 906 women with HDP between 1 April 2010 and 31 December 2014 after excluding women with pre‐existing hypertension, women aged < 12 years and > 57 years and women who died during the delivery stay. Among these, 35 878 (33%) received at least one antihypertensive treatment in the month following maternity discharge, and 73 028 did not receive any antihypertensive treatment in the month following maternity discharge. Treated women were more likely to have preeclampsia (67%), while non‐treated women had nearly as much preeclampsia as gestational hypertension. Compared to non‐treated women, women who were treated were slightly older, had a lower gestational age at delivery and had significantly higher frequencies of multiple pregnancy and of pre‐existing diabetes and dyslipidaemia in the year preceding pregnancy (Table 1). Smoking was significantly slightly more frequent in non‐treated women than in treated women. Regarding the social environment, we found that, overall, the deprivation index was slightly higher among non‐treated women, which could indicate that they lived in more disadvantaged areas than treated women, although the difference was very slight. The description of the matched cohort is presented in Table S3.

**TABLE 1:** Characteristics of women who gave birth in France between 1 April 2010 and 31 December 2014, according to treatment or non‐treatment with antihypertensive drugs at maternity discharge^a^.

	Non‐treated women *N* = 73 028	Treated women *N* = 35 878	*p*	|SMD|
	*n* (%)	*n* (%)		
*Variables during inclusion pregnancy*				
Preeclampsia	36 098 (49.43)	24 139 (67.28)	< 0.0001	0.37
Gestational hypertension	36 930 (50.57)	11 739 (32.72)		
Maternal age				
Mean ± SD	29.61 ± 5.83	30.98 ± 5.92	< 0.0001	0.23
Median (Q1‐Q3)	29 (25–34)	31 (27–35)		
< 20 years	2289 (3.13)	670 (1.87)	< 0.0001	
20–29 years	35 329 (48.38)	14 480 (40.36)		
30–39 years	31 459 (43.08)	17 707 (49.35)		
≥ 40 years	3951 (5.41)	3021 (8.42)		
Gestational age at delivery				
Mean ± SD	37.81 ± 2.84	36.43 ± 3.51	< 0.0001	0.43
Median (Q1–Q3)	38 (37–40)	37 (35–39)		
Multiple pregnancy	4130 (5.66)	2318 (6.46)	< 0.0001	0.03
Gestational diabetes	9518 (13.03)	4615 (12.86)	0.4318	0.01
Morbid obesity	1787 (2.45)	815 (2.27)	0.0748	0.01
Severe alcoholism	49 (0.07)	22 (0.06)	0.7255	0.00
Personal history of CVD	203 (0.28)	117 (0.33)	0.1678	0.01
Family history of CVD	156 (0.21)	77 (0.21)	0.9732	0.00
*Variables during inclusion pregnancy or in the year preceding pregnancy*				
Smoking	4668 (6.39)	1889 (5.27)	< 0.0001	0.05
*Variables during the year preceding pregnancy*				
Pre‐existing diabetes	1321 (1.81)	813 (2.27)	< 0.0001	0.03
Dyslipidaemia	496 (0.68)	309 (0.86)	0.001	0.02
*Information relating to social environment*				
Deprivation index				
Not available	5587 (7.65)	2563 (7.14)		
Mean ± SD	−0.08 ± 1.44	−0.14 ± 1.52	< 0.0001	0.04
Median (Q1–Q3)	0.03 ((−0.89)‐0.88)	−0.06 ((−1.05)‐0.90)		

Abbreviations: CVD, cardiovascular disease; SMD, standardised mean difference.

^a^ Women were considered treated if they had received at least one antihypertensive treatment in the month following maternity discharge. Women were considered non‐treated if they had not received any antihypertensive treatment in the month following maternity discharge.

### 3.1 Absolute Risk and Global Relative Hazard of Cardiovascular Events

In the 10 years following childbirth, nearly 1 in 5 non‐treated women developed chronic hypertension (17.99%, Table 2). For women who received antihypertensive medication in the early postpartum period, this proportion was twice as high (43.58%). The figures were similar for the composite event including chronic hypertension (Kaplan–Meier survival curves and number‐at risk tables presented in Figure S4). For the composite event excluding chronic hypertension, we also find an absolute risk higher among treated women (1.70% vs. 1.09%, Table 2).

**TABLE 2:** Occurrence of cardiovascular events among treated versus non‐treated women^a^, and effects of the antihypertensive treatment at maternity discharge^a^ on the occurrence of cardiovascular events over a 10‐year follow‐up period shown as HR [95% CI] compared to non‐treated women. Peripheral artery disease not shown as too few events for multivariate analysis.

	Number of events		Effect of antihypertensive treatment at maternity discharge^a^ during 10 years of follow‐up			
			Unmatched cohort		Matched^c^ cohort	
	Non‐treated women *n* (%)	Treated women *n* (%)	Unadjusted effect from Cox model HR [95% CI]	Adjusted^d^ effect from Cox model aHR [95% CI]	Unadjusted effect from Cox model HR [95% CI]	Adjusted^e^ effect from Cox model aHR [95% CI]
Composite event^b^ including chronic hypertension	13 432 (18.39)	15 756 (43.92)	3.111 [3.040–3.183]	3.025 [2.956–3.096]	3.547 [3.428–3.670]	3.442 [3.322–3.565]
Composite event^b^ excluding chronic hypertension	797 (1.09)	609 (1.70)	1.560 [1.404–1.734]	1.451 [1.305–1.614]	1.445 [1.276–1.637]	1.343 [1.172–1.538]
Chronic hypertension	13 139 (17.99)	15 637 (43.58)	3.154 [3.082–3.228]	3.067 [2.996–3.139]	3.604 [3.482–3.730]	3.496 [3.374–3.623]
Heart failure	171 (0.23)	152 (0.42)	1.812 [1.456–2.254]	1.682 [1.351–2.096]	1.678 [1.292–2.178]	1.537 [1.161–2.034]
Coronary heart disease	206 (0.28)	161 (0.45)	1.593 [1.296–1.958]	1.436 [1.167–1.767]	1.524 [1.191–1.949]	1.487 [1.100–2.010]
Cerebrovascular disease	411 (0.56)	301 (0.84)	1.493 [1.287–1.732]	1.406 [1.210–1.632]	1.422 [1.192–1.696]	1.312 [1.090–1.580]

Abbreviations: aHR, adjusted hazard ratio; CI, confidence interval.

^a^ Women were considered treated if they had received at least one antihypertensive treatment in the month following maternity discharge. Women were considered non‐treated if they had not received any antihypertensive treatment in the month following maternity discharge.

^b^ Defined by the occurrence of at least one of the following cardiovascular events.

^c^ Matched on propensity score calculated based on the assessment of preterm HDP, severe HDP and use of prenatal antihypertensive treatment.

^d^ Adjusted on maternal age, multiple pregnancy, gestational diabetes, morbid obesity, severe alcoholism, personal or family history of cardiovascular disease, smoking, pre‐existing diabetes and dyslipidaemia.

^e^ Adjusted on maternal age, gestational diabetes, morbid obesity, severe alcoholism, personal or family history of cardiovascular disease, smoking, pre‐existing diabetes and dyslipidaemia for composite event including chronic hypertension and for chronic hypertension; adjusted on maternal age, gestational diabetes, morbid obesity, smoking, pre‐existing diabetes and dyslipidaemia for other events because too few numbers for adjustment.

To study the relative hazard over 10 years of developing cardiovascular events for women who received antihypertensive treatment in the first postpartum month, we applied unadjusted and adjusted Cox models (maternal age, multiple pregnancy, gestational diabetes, morbid obesity, severe alcoholism, personal or family history of CVD, smoking, pre‐existing diabetes and dyslipidaemia were included in the adjustment). We found that women who received antihypertensive medication in the early postpartum period were at highest risk of long‐term cardiovascular complications (Table 2). In particular, for chronic hypertension, we observed a threefold increase in risk (aHR = 3.067, 95% CI [2.996–3.139] for the unmatched cohort). The increase was similar for the composite event including chronic hypertension (aHR = 3.025, 95% CI [2.956–3.096] for the unmatched cohort). For the composite event excluding chronic hypertension, we also found that treated women had a higher risk than non‐treated women (aHR = 1.451, 95% CI [1.305–1.614] for the unmatched cohort). For heart failure, the risk was 1.7 higher (aHR = 1.682, 95% CI [1.351–2.096] for the unmatched cohort), while for coronary heart and cerebrovascular diseases the risk was around 1.4 higher (aHR = 1.436, 95% CI [1.167–1.767] and aHR = 1.406, 95% CI [1.210–1.632], respectively, for the unmatched cohort). When women were matched on the propensity score calculated based on the assessment of preterm HDP, severe HDP and use of prenatal antihypertensive treatment, we observed results very similar to those observed for the unmatched cohort (Table 2).

Besides, we observed, as expected for all variables considered a risk factor, a higher risk of cardiovascular events (Table S5 for the unmatched cohort, Table S6 for the matched cohort). In contrast, women with multiple pregnancies appear to be less predisposed to postpartum and long‐term cardiovascular disease than women with a single pregnancy (for the unmatched cohort, composite event including chronic hypertension: aHR = 0.563, 95% CI [0.532–0.597], composite event excluding chronic hypertension: aHR = 0.724, 95% CI [0.568–0.925] and chronic hypertension: aHR = 0.560, 95% CI [0.528–0.593]).

### 3.2 Time Trends

We then applied the extended Cox Step Function model to estimate the effect of the antihypertensive treatment in different time intervals, from 3 months following maternity discharge, between 3 and 6 months, between 6 months and 1 year and annually thereafter (Table 3). We found that for chronic hypertension, treated women have a more than 100‐fold higher risk within 3 months of maternity discharge (aHR = 141.968, 95% CI [111.571–180.646]), and only a 1.3‐fold higher risk after 10 years (1.313, 95% CI [1.189–1.450]). The same relative hazard difference was observed over the decade following childbirth for the composite event including chronic hypertension. For the composite event excluding chronic hypertension, we observed a similar trend, with the highest relative hazard within 3 months of maternity discharge (aHR = 3.655, 95% CI [2.352–5.680]). We observed greater fluctuations for this event due to smaller sample sizes, resulting in much wider and overlapping confidence intervals. However, in the tenth and final year of follow‐up, we observed an aHR = 1.325, 95% CI [1.006–1.745]. Finally, the RMST analysis showed that treated women had events earlier than non‐treated women (Table S7).

**TABLE 3:** Time intervals^a^ adjusted^b^ effects of antihypertensive treatment at maternity discharge^c^ on the occurrence of cardiovascular events over a 10‐year follow‐up period.

Time intervals	Composite event^d^ including chronic hypertension	Composite event^d^ excluding chronic hypertension	Chronic hypertension
	aHR [95% CI]	aHR [95% CI]	aHR [95% CI]
Before 3 months	107.472 [87.145–132.541]	3.655 [2.352–5.680]	141.968 [111.571–180.646]
3–6 months	15.563 [14.144–17.124]	0.788 [0.377–1.649]	16.057 [14.576–17.689]
6 m‐1 year	3.314 [3.048–3.602]	0.835 [0.455–1.533]	3.361 [3.090–3.655]
1–2 years	1.686 [1.555–1.828]	1.480 [0.993–2.207]	1.676 [1.545–1.819]
2–3 years	1.692 [1.558–1.838]	1.705 [1.140–2.551]	1.698 [1.563–1.846]
3–4 years	1.638 [1.505–1.783]	1.007 [0.664–1.526]	1.665 [1.528–1.813]
4–5 years	1.400 [1.279–1.532]	1.137 [0.812–1.594]	1.410 [1.287–1.544]
5–6 years	1.469 [1.337–1.615]	1.671 [1.177–2.372]	1.468 [1.334–1.615]
6–7 years	1.522 [1.381–1.677]	1.013 [0.713–1.438]	1.548 [1.404–1.707]
7–8 years	1.352 [1.226–1.491]	1.426 [1.056–1.924]	1.357 [1.229–1.498]
8–9 years	1.432 [1.295–1.583]	2.264 [1.643–3.119]	1.418 [1.282–1.569]
9–10 years	1.311 [1.189–1.447]	1.325 [1.006–1.745]	1.313 [1.189–1.450]

Abbreviations: aHR, adjusted hazard ratio; CI, confidence interval.

^a^ Extended Cox regression with step function multivariate model.

^b^ Adjusted on maternal age, multiple pregnancy, gestational diabetes, morbid obesity, severe alcoholism, personal or family history of CVD, smoking, pre‐existing diabetes and dyslipidaemia.

^c^ Women were considered treated if they had received at least one antihypertensive treatment in the month following maternity discharge. Women were considered non‐treated if they had not received any antihypertensive treatment in the month following maternity discharge.

^d^ Defined by the occurrence of at least one cardiovascular event.

### 3.3 Sensitivity Analyses

All sensitivity analyses (first one including only women aged 15–49, second one including only women for whom we had access to the deprivation index, and third one excluding women with preterm or severe HDP) yielded results similar to those of the main analyses (Tables S8–S10).

## 4 Discussion

This study benefits from a large, nationally representative cohort with near‐complete capture of births and longitudinal linkage, offering real‐world insights into routine clinical care. In addition, we use robust modelling methods, such as RMST which does not require the assumption that risks are proportional, and an extended Cox regression with a step function model which allowed us to examine dynamic postnatal risk, accounting for non‐proportional hazards. This powerful and flexible approach enabled a comprehensive assessment of cardiovascular and cerebrovascular events, strengthening the reliability of our findings.

Our study confirms that previously normotensive women who receive antihypertensive medication in the early postpartum period have the highest risk of persistent hypertension and other cardiovascular complications. These findings persist even after propensity score analysis based on preterm HDP, clinically severe HDP and use of prenatal antihypertensive treatment. The study findings also indicate that treated women had events earlier than non‐treated women, presenting a higher risk at the beginning of the postpartum period. By year 10, and particularly from year 7 onward, the risk in the treated group approached that of the non‐treated group. This convergence should be interpreted with caution, as it may reflect differences in baseline risk, natural disease trajectories, rather than a causal effect of treatment, although a potential contribution of treatment cannot be excluded.

### 4.1 Clinical Implications

The study findings highlight two key concerns regarding postpartum antihypertensive management. Firstly, despite taking antihypertensive medications for at least the first month postpartum, more than 40% of these women progress to develop chronic hypertension, suggesting limited long‐term effectiveness of treatment. Secondly, nearly 20% of women with HDP that were not treated in the first postpartum month developed chronic hypertension within 10 years, suggesting that the current clinical criteria used to initiate antihypertensive treatment insufficiently captures maternal cardiovascular risk. Consistent with this assertion, only 33% of HDP women received antihypertensive medication postpartum: a proportion that appears insufficient given the high incidence of chronic hypertension observed over the subsequent decade. The fact that most physicians and many women hold a misconception that HDP consequences are temporary and resolve after birth may have contributed to underestimation of postpartum cardiovascular risks [27]. These findings indicate that high‐risk women are not being identified early and may be missing opportunities for timely intervention and highlights the need for improved risk stratification tools to better identify women to benefit from early intervention, as well as standardised treatment pathways that include close monitoring and individualised titration of therapy [28].

The POP‐HT trial demonstrated the importance of early and effective postpartum antihypertensive treatment for 3 months in all women after HDP [13]. Our nationwide data support the clinical effectiveness of POP‐HT trial findings, whilst concurrently demonstrating the detrimental cardiovascular impact of maternal undertreatment. This highlights the critical gaps in current postpartum hypertension care in France and perhaps also globally. The failure to detect and treat nearly one in five women who later develop chronic hypertension underscores the limitations of current clinical protocols that rely solely on blood pressure levels in the early postpartum period to guide antihypertensive treatment decisions. Conversely, initiating treatment in high‐risk women without structured follow‐up and titration, as undertaken in the POP‐HT trial, may not be sufficient to achieve long‐term cardiovascular protection in these women. During the period covered by our study, there were no specific national guidelines regarding antihypertensive treatment in the postpartum period. As a consequence, antihypertensive use during pregnancy was traditionally limited to cases of markedly elevated blood pressure (BP ≥ 160/110 mmHg), reflecting a restrictive therapeutic approach [29]. However, following the publication of a randomised controlled trial [30], French CNGOF 2023 clinical practice guidelines revised the thresholds for initiating antihypertensive therapy during pregnancy, recommending treatment at lower blood pressure levels (≥ 140/90 mmHg) than previously advised [31]. It is plausible that this broader use of antihypertensive agents during pregnancy influenced prescribing habits in the postpartum period, even though formal postpartum recommendations were lacking.

An unexpected study finding was that HDP women with multiple pregnancies who received antihypertensive treatment had a 40% lower risk of cardiovascular complications. This novel finding may reflect the distinct pathophysiology of HDP in multiple gestations. In HDP, pregnancy is seen as a stress test that unmasks an underlying maternal cardiovascular vulnerability, explaining the predisposition to long‐term cardiovascular disease. In the case of multiple pregnancies, the development of HDP may be driven more by the increased haemodynamic demands of the multiple gestations rather than by pre‐existing cardiovascular risk, offering a potential explanation for reduced postpartum cardiovascular complications [32].

### 4.2 Research Implications

Given that the study findings support the antihypertensive treatment protocols used in the POP‐HT trial [13], it is appropriate to surmise that the current standard of care limited to a midwife consultation at 10 days and a physician visit at 8 weeks is insufficient. New models of care should combine identification of at‐risk women along with structured protocols for initiating, monitoring and adjusting antihypertensive therapy [33]. Further research should investigate the optimal timing, drug choice and duration of postpartum antihypertensive treatment [34, 35, 36, 37, 38, 39, 40]. Studies should also evaluate whether integrated follow‐up pathways involving primary care, obstetricians, cardiologists, midwives and self‐care interventions can improve adherence and long‐term outcomes [41, 42]. Our findings provide a rationale and a benchmark for designing such prospective interventional studies.

### 4.3 Strengths and Limitations

The strengths of our study include its large, nationally representative cohort with near‐complete capture of births, longitudinal linkage and reliance on real‐world data, allowing insight into routine clinical care. Moreover, our data are exhaustive at the national level, which strengthens the generalisability of our findings. These data are essential to assess the actual impact of interventions such as promoted by the POP‐HT trial, beyond the strict eligibility criteria and controlled conditions of randomised clinical trials. Besides, one of another strength of our analyses is that we consider the non‐proportionality of risks. Indeed, we used RMST modelling which does not assume that risks are proportional. To account for this non‐proportionality and to study the evolution of the postnatal effect of treatment over time, we used an extended Cox regression with a step function model. This method of modelling can provide an estimate in disjointed time intervals, allowing estimates of the effect in each interval separately considering the deviation from proportionality, which would not have been possible using the traditional proportional hazard Cox model. In addition, this Cox regression with a step function model allows us to gain power as these estimates are obtained in a single model. This robust approach allowed outputs for all cardiovascular and cerebrovascular events.

However, our analysis is subject to the limitations inherent in administrative datasets. First, we unfortunately do not have data on ethnicity for each patient in France although this is a relevant issue in this context [43]. Furthermore, some information may be incomplete as coding practices may vary from one institution to another, even if quality improvements are obtained through internal and external validations. It can nevertheless be assumed that, despite this, there is still potential for coding errors and comorbidities and risk factors may not always be fully documented in the PMSI database. For example, we can assume that smoking is only noted in cases of heavy smoking, and this is why we tried to better define this variable by looking for the prescription of nicotine substitutes. In addition, a previous validation study confirmed the quality and exhaustiveness of PMSI data for perinatal algorithms [44]. Regardless, even if some data may be incomplete, this should not differ between the two groups compared, since the information is collected in the same way for all women. In addition, we were only able to include BMIs ≥ 40 kg/m^2^ because light to moderate obesity can sometimes be poorly reported, particularly among pregnant women for whom it can be difficult to obtain pre‐pregnancy weight. Thus, although the link between obesity and risk of hypertension or long‐term cardiovascular disease is established even for BMIs < 40 kg/m^2^, the cut‐off of BMI ≥ 40 kg/m^2^ was chosen to ensure that the cases represented confirmed obesity. Another point is that we do not know the reasons why some women were treated and others were not, assuming only that the treatment was in line with national recommendations at the time of the study [30]. Also, we defined treatment exposure based on pharmacy dispensing data, which may not accurately reflect patient adherence and compliance. Our approach assumed that earlier initiation could offer greater benefit, but the optimal treatment window remains uncertain. In addition, our definition of chronic hypertension, based on three consecutive antihypertensive prescriptions, may lead to earlier classification among women treated in the immediate postpartum period. This definition was chosen because the standard antihypertensive management protocol following HDP was for a one‐month prescription at discharge from hospital following birth. The women who then received two additional months of antihypertensive medication would have done so because of a proactive medical decision by the family practitioner—almost always for persistent hypertension. As actual postnatal blood pressure measurements are not available on our database, the definition we used seems to be the best proxy for persistent postnatal hypertension. To further minimise the risk of misclassifying pre‐existing chronic hypertension, we excluded women who received antihypertensive treatment in the 6 months preceding pregnancy, during the first 19 weeks of pregnancy and were hospitalised for chronic hypertension during pregnancy. Finally, despite the propensity score analyses, the observational nature of the study introduces potential confounding by indication, as treated women likely had more severe hypertensive disease at baseline.

## 5 Conclusion

Despite the potential benefit of early antihypertensive treatment in the postpartum period after HDP, chronic hypertension occurs in 44% of these women in the 10 years following pregnancy. The study also demonstrated that chronic hypertension is seen in 18% of women who were not treated with antihypertensives. These findings support the need for new evidence‐based strategies to guide postpartum hypertension management, including surveillance protocols and treatment pathways. As clinical practices evolve, particularly considering recent trial data, our study provides a benchmark against which to assess future improvements in postpartum cardiovascular care.

## Author Contributions

E.S., B.T. and C.Q. were involved in the conception and design of the study. C.Q. was the coordinator of the study. S.T., J.C. and C.Q. were responsible for the data collection. E.S., S.T., B.T. and C.Q. wrote the first draft. S.T. was in charge of the analysis. S.T., J.C. and C.Q. accessed and verified the data. E.S., B.T., S.B.‐Q. and C.Q. were involved in the interpretation. All the authors contributed to the critical revision of the manuscript and approved the final version.

## Funding

This work was supported by the Agence Régionale de Santé Bourgogne Franche Comté, whose commitment we gratefully acknowledge.

## Ethics Statement

This study was approved by the French National Committee for Data Protection (declaration of conformity to the methodology of reference 05 obtained on 7/08/2018 under the number 2204633 v0).

## Consent

Written consent was not needed for this study as it was a retrospective study and that the national data used were anonymous.

## Conflicts of Interest

The authors declare no conflicts of interest.

## Supporting information


**FIGURE S1:** Study flow chart.
**FIGURE S4:** Kaplan–Meier survival curves and number‐at risk tables among treated and non‐treated women for the cardiovascular events.
**TABLE S2:** ICD‐10 and CCMP codes, LTIs and ATC classes considered.
**TABLE S3:** Characteristics of women after matching on propensity score**, according to treatment or non‐treatment with antihypertensive drugs at maternity discharge*.
**TABLE S5:** Adjusted effects and absolute risks of covariates on the occurrence of cardiovascular events over a 10‐year follow‐up period for the unmatched cohort, traditional Cox model.
**TABLE S6:** Adjusted effects of covariates on the occurrence of cardiovascular events over a 10‐year follow‐up period for the matched cohort^$^, traditional Cox model.
**TABLE S7:** Adjusted difference in Restricted Mean Survival Time (RMST) between treated* and non‐treated women regarding cardiovascular events for unmatched and matched^$^ cohorts.
**TABLE S8:** Sensitivity analysis including only women aged 15–49: Absolute risks, traditional Cox model and extended Cox regression with a step function model (unmatched cohort).
**TABLE S9:** Sensitivity analysis including only women for whom we had access to the deprivation index: Absolute risks, traditional Cox model and extended Cox regression with a step function model (unmatched cohort).
**TABLE S10:** Sensitivity analysis excluding women with preterm or severe hypertensive disorders of pregnancy: Absolute risks, traditional Cox model and extended Cox regression with a step function model (unmatched cohort).

## Data Availability

The database was transmitted by the National Health Insurance (CNAM‐Caisse nationale de l'assurance maladie), responsible and owner for the ‘National System of Health Data’ (SNDS) database. The use of these data by our department was approved by the National Committee for data protection. We are not allowed to transmit these data. Data used in this study are available for researchers who meet the criteria for access to these French data from the National Health Insurance (training that opens a personal accreditation, approval of the protocol by required authorities (Ethics and Scientific Committee for Research, Studies and Evaluations in the Field of Health‐CESREES and National Committee for data protection‐CNIL)) according to the ‘Décret no. 2016‐1872 du 26 décembre 2016 modifiant le décret no. 2005‐1309 du 20 octobre 2005 pris pour l'application de la loi no. 78‐17 du 6 janvier 1978 relative à l'informatique, aux fichiers et aux libertés’ (https://www.legifrance.gouv.fr/eli/decret/2016/12/26/2016‐1872/jo/texte). A permanent access is granted to some public services within the limits established by laws (Décret no. 2016‐1871 du 26 décembre 2016) relatif au traitement de données à caractère personnel dénommé ‘système national des données de santé’ (https://www.legifrance.gouv.fr/eli/decret/2016/12/26/2016‐1871/jo/texte).
