# Supplementary material for: Long‐Term Cardiovascular Impact of Postpartum Treatment After Hypertensive Disorders of Pregnancy: Population‐Based Cohort Study
Source: BJOG. 2026 Apr 22;133(11):2003–12. doi: 10.1111/1471-0528.70251 (PMC13571887; doi:10.1111/1471-0528.70251)
Supplement: Supplementary file 1 [file BJO-133-2003-s001.docx]

**FIGURE S1.** Study Flow chart.

All women's pregnancies between April 1, 2010 and December 31, 2014

**N = 3 788 230**

Pregnancies with a diagnosis of pre-eclampsia or gestational hypertension

**N = 131 435**

First pregnancy of the inclusion period

**N = 127 384**

Exclusion criteria:

- Delivery of antihypertensive treatment in the 6 months preceding pregnancy or during the 19 first weeks of pregnancy *(n=13 414 (10%))*

**N = 113 970**

- Hospitalisation for chronic hypertension during pregnancy *(n=5 045 (4%))*

**N = 108 925**

- Maternal age <12 or >55 years *(n =1 (0%))*

**N = 108 924**

- Death during the delivery stay *(n = 18 (0%))*

**N = 108 906**

Final study cohort of women with hypertensive disorders of pregnancy

**N = 108 906**

**Treated women**

**N = 35 878**

**Non-treated women**

**N = 73 028**

**TABLE S2.** ICD-10 and CCMP codes, LTIs and ATC classes considered.

| **Cardiovascular events (ICD-10 and CCMP codes)** | |
| --- | --- |
| **Chronic hypertension** | I10-I13, I15 |
| **Heart failure** | I50 |
| **Coronary heart disease** |  |
| Angina pectoris | I20 |
| Myocardial infarction | I21-I23 |
| Coronary revascularization  *(CCMP codes)* | DDAF001 DDAF006 DDAF004 DDAF003 DDAF010 DDAF008 DDAF007 DDAF009 DDMA025 DDMA015 DDMA023 DDMA017 DDMA032 DDMA011 DDMA029 DDMA018 DDMA038 DDMA021 DDMA026 DDMA020 DDMA031 DDMA006 DDMA033 DDMA008 DDMA022 DDMA005 DDMA034 DDMA009 DDMA030 DDMA003 DDMA035 DDMA013 DDMA036 DDMA012 DDMA028 DDMA007 DDMA024 DDMA019 DDMA027 DDMA016 DDMA037 DDMA004 DDPF002 DDFF002 DDFF001 DDAA002 |
| **Cerebrovascular disease** |  |
| Ischemic and haemorrhagic stroke | I60-I61, I62.9, I63-I64 |
| Transient ischemic attacks | G45 except G45.4, G46 |
| Carotid revascularization  *(CCMP codes)* | EBFA005 EBFA003 EBFA010 EBCA017 EBKA001 EBKA003 EBFA021 EBFA012 EBFA006 EBFA016 EBFA002 EBFA008 EBFA015 EBKA004 EBFA019 EBFA014 EBFA009 EBKA002 EBFA013 |
| **Peripheral artery disease** |  |
| Peripheral artery disease | I70.2, I70.8-9, I74.3-I74.4 |
| Arterial revascularization  *(CCMP codes)* | EDAF002 EDAF003 DGPF001 DGPF002 EDPF008 EDPF009 EDPF006 DGFA010 DGFA004 DGFA012 DGFA009 DGFA003 DGFA007 DGFA008 EDFA006 EDFA003 EDFA007 DGCA019 DGCC859 DGCA009 DGCC822 DGCA026 DGCC857 DGCA022 DGCC862 DGCA010 DGCC825 DGCA004 DGCC846 DGCA030 DGCC810 DGCA020 DGCC872 DGCA029 DGCC835 EDCA003 EEAF003 EEAF004 EEAF005 EEAF002 EEAF001 EEAF006 EENF002 EENF001 EEPF002 EEPF001 EEJF001 EEFA004 EEFA002 EEFA001 EEFA003 EECA007 EECA006 EDCA005 EDCA004 EECA002 EECA001 EECA003 EECA008 EECA010 EECA005 EECA012 EEGA001 EEGA002 EEAA002 ENAF002 ENAF001 ENNF001 ENFF001 ENFA006 ENFA001 ENFA004 ENFA005 DGLF002 DGLF001 DGLF005 if ICD-10 codes I70.2, I74.3, I74.5 |
| **Treatments (ATC classes)** | |
| **Antihypertensive drugs** | C02AB02, C02AC01, C02AC02, C02AC05, C02AC06, C02CA01, C02CA06, C02DC01, C02LA01, C03AA01, C03AA03, C03BA04, C03BA10, C03BA11, C03BX03, C03CA01, C03CA02, C03CA03, C03DA01, C03DB01, C03EA01, C03EA04, C07AA02, C07AA03, C07AA05, C07AA06, C07AA12, C07AA15, C07AA16, C07AA23, C07AB02, C07AB03, C07AB04, C07AB05, C07AB07, C07AB08, C07AB12, C07AG01, C07BA02, C07BB02, C07BB03, C07BB07, C07BB12, C07CA03, C07DA06, C07FB02, C07FB03, C08CA01, C08CA02, C08CA03, C08CA04, C08CA05, C08CA08, C08CA09, C08CA11, C08CA13, C08CX01, C08DA01, C08DB01, C08GA02, C09AA01, C09AA02, C09AA03, C09AA04, C09AA05, C09AA06, C09AA07, C09AA08, C09AA09, C09AA10, C09AA13, C09AA15, C09AA16, C09BA01, C09BA02, C09BA03, C09BA04, C09BA05, C09BA06, C09BA07, C09BA09, C09BA15, C09BB02, C09BB04, C09BB07, C09BB10, C09BX02, C09CA01, C09CA02, C09CA03, C09CA04, C09CA06, C09CA07, C09CA08, C09DA01, C09DA02, C09DA03, C09DA04, C09DA06, C09DA07, C09DA08, C09DB01, C09DB02, C09DB04, C09XA02, C09XA52, C10BX03 |
| **Nicotine substitutes** | N07BA, N06AX12 |
| **Antidiabetic drugs** | A10 |
| **Hypolipidemics** | C10 |
| **Other variables (ICD-10 codes)** | |
| **Gestational diabetes** | O24 |
| **Morbid obesity  (BMI ≥40 kg/m^2^)** | E66.01-02, E66.06-07, E66.11-12, E66.16-17, E66.21-22, E66.26-27, E66.81-82, E66.86-87, E66.91-92, E66.96-97 |
| **Smoking** | F17 (except F17.0), T65.2, Z72.0 |
| **Severe alcoholism** | F10 (except F10.0), G62.1, G72.1, I42.6, K29.2, K85.2, K86.0, K70 |
| **Family or personal history of cardiovascular disease** | I69, Z86.7, Z82.4 |
| **Pre-existing diabetes** | E10-E14 |
| **Dyslipidemia** | E78 |
| **Long-term illnesses^*^** | |
| **Diabetes** | 8*: Type 1 diabetes and type 2 diabetes in adults or children* |

*^*^* *Long-Term Illnesses (LTIs) entitle patients to coverage without coinsurance under the French national health insurance program*

**TABLE S3.** Characteristics of women after matching on propensity score^**^, according to treatment or non-treatment with antihypertensive drugs at maternity discharge^*^**.**

|  | **Non-treated women** | **Treated women** | **p value** | **\|SMD ^µ^\|** |
| --- | --- | --- | --- | --- |
|  | **N = 35 756** | **N = 35 756** |  |  |
|  | **n (%)** | **n (%)** |  |  |
| **Variables during inclusion pregnancy** |  |  |  |  |
| **Preeclampsia** | 20 004 (55.95) | 24 017 (67.17) | <.0001 | 0.23 |
| **Gestational hypertension** | 15 752 (44.05) | 11 739 (32.83) |  |  |
| **Maternal age** |  |  |  |  |
| **Mean +/- SD** | 29.64 +/- 5.83 | 30.98 +/- 5.92 | <.0001 | 0.23 |
| **Median (Q1-Q3)** | 29 (26-34) | 31 (27-35) |  |  |
| **<20 years** | 1 068 (2.99) | 668 (1.87) | <.0001 |  |
| **20-29 years** | 17 337 (48.49) | 14 431 (40.36) |  |  |
| **30-39 years** | 15 366 (42.97) | 17 651 (49.37) |  |  |
| **>=40 years** | 1 985 (5.55) | 3 006 (8.41) |  |  |
| **Gestational age at delivery** |  |  |  |  |
| **Mean +/- SD** | 36.84 +/- 3.36 | 36.45 +/- 3.50 | <.0001 | 0.12 |
| **Median (Q1-Q3)** | 38 (35-39) | 37 (35-39) |  |  |
| **Multiple pregnancy** | 2 989 (8.36) | 2 304 (6.44) | <.0001 | 0.07 |
| **Gestational diabetes** | 4 680 (13.09) | 4 597 (12.86) | 0.3556 | 0.01 |
| **Morbid obesity** | 892 (2.49) | 814 (2.28) | 0.056 | 0.01 |
| **Severe alcoholism** | 29 (0.08) | 22 (0.06) | 0.3268 | 0.01 |
| **Personal history of CVD^§^** | 113 (0.32) | 117 (0.33) | 0.7916 | 0.00 |
| **Family history of CVD^§^** | 72 (0.20) | 77 0.22) | 0.6818 | 0.00 |
| **Variables during inclusion pregnancy or in the year preceding pregnancy** | | |  |  |
| **Smoking** | 2 292 (6.41) | 1 881 (5.26) | <.0001 | 0.05 |
| **Variables during the year preceding pregnancy** | |  |  |  |
| **Pre-existing diabetes** | 721 (2.02) | 813 (2.27) | 0.0176 | 0.02 |
| **Dyslipidaemia** | 257 (0.72) | 308 (0.86) | 0.0312 | 0.02 |
| **Information relating to social environment** | |  |  |  |
| **Deprivation index** |  |  |  |  |
| ***Not available*** | *2 819 (7.88)* | *2 561 (7.16)* |  |  |
| **Mean +/- SD** | -0.08 +/- 1.44 | -0.14 +/- 1.52 | <.0001 | 0.04 |
| **Median (Q1-Q3)** | 0.02 ((-0.89)-0.88) | -0.06 ((-1.05)-0.90) |  |  |

*^§^ CVD = cardiovascular disease*

*^*^ Women were considered treated if they had received at least one antihypertensive treatment in the month following maternity discharge. Women were considered non-treated if they had not received any antihypertensive treatment in the month following maternity discharge*

*^**^* *Propensity score* *calculated based on the assessment of preterm HDP, severe HDP, and use of prenatal antihypertensive treatment*

*^µ^ SMD = standardized mean difference*

**FIGURE S4.** Kaplan-Meier survival curves and number-at risk tables among treated and non-treated women for the cardiovascular events.

**TABLE S5.** Adjusted effects and absolute risks of covariates on the occurrence of cardiovascular events over a 10-year follow-up period for the unmatched cohort, traditional Cox model.

|  |  | **Composite event^µ^ including chronic hypertension** | **Composite event^µ^ excluding chronic hypertension** | **Chronic hypertension** | **Heart failure** | **Coronary heart disease** | **Cerebrovascular disease** |
| --- | --- | --- | --- | --- | --- | --- | --- |
|  |  | **aHR [95%CI]** | **aHR [95%CI]** | **aHR [95%CI]** | **aHR [95%CI]** | **aHR [95%CI]** | **aHR [95%CI]** |
| **Treatment^*^** | ***n (%)*** | 15 756 (43.92) | 609 (1.70) | 15 637 (43.58) | 152 (0.42) | 161 (0.45) | 301 (0.84) |
|  | ***aHR [95%CI]*** | 3.025 [2.956 - 3.096] | 1.451 [1.305 - 1.614] | 3.067 [2.996 - 3.139] | 1.682 [1.351 - 2.096] | 1.436 [1.167 - 1.767] | 1.406 [1.210 - 1.632] |
| **Age (ref <20 years)** |  |  |  |  |  |  |  |
| **20-29 years** | ***n (%)*** | 10 231 (20.54) | 411 (0.83) | 10 064 (20.21) | 89 (0.18) | 79 (0.16) | 230 (0.46) |
|  | ***aHR [95%CI]*** | 2.419 [2.135 - 2.740] | 3.369 [1.596 - 7.112] | 2.434 [2.146 - 2.762] | 2.475 [0.609 - 10.055] | 2.274 [0.559 - 9.255] | 4.488 [1.437 - 14.019] |
| **30-39 years** | ***n (%)*** | 15 765 (32.06) | 800 (1.63) | 15 560 (31.65) | 189 (0.38) | 216 (0.44) | 397 (0.81) |
|  | ***aHR [95%CI]*** | 3.860 [3.408 - 4.370] | 6.313 [2.998 - 13.292] | 3.892 [3.432 - 4.413] | 4.921 [1.220 - 19.846] | 5.896 [1.464 - 23.753] | 7.667 [2.461 - 23.886] |
| **>=40 years** | ***n (%)*** | 2 939 (42.15) | 188 (2.70) | 2 905 (41.67) | 43 (0.62) | 70 (1.00) | 82 (1.18) |
|  | ***aHR [95%CI]*** | 5.103 [4.487 - 5.804] | 9.809 [4.608 - 20.884] | 5.145 [4.517 - 5.861] | 7.272 [1.757 - 30.101] | 12.344 [3.020 - 50.453] | 10.728 [3.385 - 34.001] |
| **Multiple pregnancy** | ***n (%)*** | 1 203 (18.66) | 68 (1.05) | 1 179 (18.28) | 18 (0.28) | 14 (0.22) | 33 (0.51) |
|  | ***aHR [95%CI]*** | 0.563 [0.532 - 0.597] | 0.724 [0.568 - 0.925] | 0.560 [0.528 - 0.593] | 0.841 [0.523 - 1.355] | 0.547 [0.321 - 0.934] | 0.698 [0.492 - 0.991] |
| **Gestational diabetes** | ***n (%)*** | 5 047 (35.71) | 312 (2.21) | 4 985 (35.27) | 79 (0.56) | 108 (0.76) | 118 (0.83) |
|  | ***aHR [95%CI]*** | 1.284 [1.244 - 1.326] | 1.397 [1.217 - 1.604] | 1.288 [1.247 - 1.330] | 1.567 [1.187 - 2.068] | 1.771 [1.378 - 2.276] | 1.111 [0.901 - 1.370] |
| **Morbid obesity** | ***n (%)*** | 1 055 (40.55) | 50 (1.92) | 1 043 (40.08) | 19 (0.73) | 11 (0.42) | 21 (0.81) |
|  | ***aHR [95%CI]*** | 1.616 [1.518 - 1.719] | 1.196 [0.898 - 1.592] | 1.620 [1.522 - 1.725] | 2.063 [1.286 - 3.310] | 0.844 [0.459 - 1.552] | 1.138 [0.734 - 1.765] |
| **Severe alcoholism** | ***n (%)*** | 26 (36.62) | 5 (7.04) | 26 (36.62) | 2 (2.82) | 2 (2.82) | 1 (1.41) |
|  | ***aHR [95%CI]*** | 1.178 [0.801 - 1.732] | 2.763 [1.141 - 6.694] | 1.208 [0.821 - 1.776] | 5.417 [1.321 - 22.215] | 2.621 [0.645 - 10.651] | 1.347 [0.188 - 9.641] |
| **Personal history of CVD^§^** | ***n (%)*** | 111 (34.69) | 18 (5.63) | 103 (32.19) | 3 (0.94) | 4 (1.25) | 7 (2.19) |
|  | ***aHR [95%CI]*** | 1.161 [0.964 - 1.400] | 3.025 [1.891 - 4.839] | 1.082 [0.891 - 1.313] | 2.177 [0.692 - 6.846] | 2.095 [0.773 - 5.675] | 2.626 [1.242 - 5.551] |
| **Family history of CVD^§^** | ***n (%)*** | 88 (37.77) | 5 (2.15) | 86 (36.91) | 1 (0.43) | 1 (0.43) | 3 (1.29) |
|  | ***aHR [95%CI]*** | 1.459 [1.184 - 1.800] | 1.280 [0.531 - 3.085] | 1.449 [1.172 - 1.790] | 1.169 [0.164 - 8.339] | 0.873 [0.122 - 6.225] | 1.660 [0.533 - 5.172] |
| **Smoking** | ***n (%)*** | 1 874 (28.58) | 182 (2.78) | 1 826 (27.85) | 34 (0.52) | 71 (1.08) | 78 (1.19) |
|  | ***aHR [95%CI]*** | 1.129 [1.078 - 1.184] | 2.285 [1.952 - 2.675] | 1.112 [1.060 - 1.166] | 1.748 [1.218 - 2.509] | 3.655 [2.812 - 4.752] | 1.943 [1.533 - 2.462] |
| **Pre-existing diabetes** | ***n (%)*** | 959 (44.94) | 99 (4.64) | 941 (44.10) | 23 (1.08) | 41 (1.92) | 25 (1.17) |
|  | ***aHR [95%CI]*** | 1.435 [1.341 - 1.536] | 2.554 [2.038 - 3.202] | 1.421 [1.327 - 1.521] | 2.342 [1.471 - 3.729] | 3.603 [2.493 - 5.207] | 1.454 [0.952 - 2.221] |
| **Dyslipidaemia** | ***n (%)*** | 367 (45.59) | 37 (4.60) | 359 (44.60) | 7 (0.87) | 12 (1.49) | 12 (1.49) |
|  | ***aHR [95%CI]*** | 1.510 [1.361 - 1.676] | 2.015 [1.436 - 2.828] | 1.491 [1.342 - 1.657] | 1.599 [0.738 - 3.463] | 1.857 [1.018 - 3.385] | 1.676 [0.937 - 2.999] |

*^*^* *Women were considered treated if they had received at least one antihypertensive treatment in the month following maternity discharge. Women were considered non-treated if they had not received any antihypertensive treatment in the month following maternity discharge*

*^µ^ Defined by the occurrence of at least one of the following cardiovascular events*

*^§^ CVD = cardiovascular disease*

*aHR=adjusted hazard ratio; CI confidence interval*

*n (%): number (percentage) of events in each subgroup*

**TABLE S6.** Adjusted effects of covariates on the occurrence of cardiovascular events over a 10-year follow-up period for the matched cohort^$^, traditional Cox model.

|  | **Composite event^µ^ including chronic hypertension** | **Composite event^µ^ excluding chronic hypertension** | **Chronic hypertension** | **Heart failure** | **Coronary heart disease** | **Cerebrovascular disease** |
| --- | --- | --- | --- | --- | --- | --- |
|  | **aHR [95% CI]** | **aHR [95% CI]** | **aHR [95% CI]** | **aHR [95% CI]** | **aHR [95% CI]** | **aHR [95% CI]** |
| **Treatment^*^** | 3.442 [3.322 - 3.565] | 1.343 [1.172-1.538] | 3.496 [3.374 - 3.623] | 1.537 [1.161 - 2.034] | 1.487 [1.100 - 2.010] | 1.312 [1.090 - 1.580] |
| **Age** | 1.056 [1.051 - 1.060] | 1.070 [1.052-1.088] | 1.056 [1.051 - 1.060] | 1.074 [1.035 - 1.113] | 1.110 [1.069 - 1.153] | 1.063 [1.039 - 1.087] |
| **Gestational diabetes** | 1.235 [1.147 - 1.330] | 1.128 [0.848-1.499] | 1.241 [1.151 - 1.337] | 1.007 [0.560 - 1.811] | 1.028 [0.561 - 1.882] | 1.030 [0.689 - 1.540] |
| **Morbid obesity** | 1.581 [1.361 - 1.836] | 1.605 [0.922-2.794] | 1.544 [1.328 - 1.794] | 1.328 [0.510 - 3.457] | 1.401 [0.434 - 4.523] | 1.487 [0.641 - 3.447] |
| **Severe alcoholism** | 1.058 [0.451 - 2.481] | - | 0.891 [0.378 - 2.101] | - | - | - |
| **Personal history of CVD^§^** | 1.037 [0.691 - 1.558] | - | 0.944 [0.622 - 1.432] | - | - | - |
| **Family history of CVD^§^** | 1.213 [0.722 - 2.038] | - | 1.131 [0.673 - 1.901] | - | - | - |
| **Smoking** | 1.072 [0.966 - 1.190] | 1.932 [1.393-2.681] | 1.056 [0.950 - 1.173] | 1.420 [0.726 - 2.774] | 4.425 [2.164 - 9.046] | 1.401 [0.879 - 2.233] |
| **Pre-existing diabetes** | 1.684 [1.427 - 1.988] | 4.407 [2.380-8.159] | 1.644 [1.392 - 1.943] | 2.976 [0.863 - 10.264] | 7.978 [2.257 - 28.194] | 2.532 [0.962 - 6.660] |
| **Dyslipidaemia** | 1.325 [1.023 - 1.715] | 1.466 [0.664-3.238] | 1.283 [0.989 - 1.664] | 1.235 [0.175 - 8.717] | 0.538 [0.124 - 2.342] | 2.036 [0.597 - 6.941] |

*^$^ Matched on the propensity score calculated based on the assessment of preterm HDP, severe HDP, and use of prenatal antihypertensive treatment*

*^*^ Women were considered treated if they had received at least one antihypertensive treatment in the month following maternity discharge. Women were considered non-treated if they had not received any antihypertensive treatment in the month following maternity discharge*

*^µ^ Defined by the occurrence of at least one cardiovascular event*

*^§^ CVD = cardiovascular disease*

*aHR = adjusted hazard ratio; CI confidence interval*

**TABLE S7.** Adjusted difference in Restricted Mean Survival Time (RMST) between treated^*^ and non-treated women regarding cardiovascular events for unmatched and matched^$^ cohorts.

|  | **Unmatched cohort** | **Matched cohort** |
| --- | --- | --- |
|  | **Adjusted^**^ rmstD***^§^* **[95%CI]** | **Adjusted^£^ rmstD***^§^* **[95%CI]** |
| **Composite event^µ^ including chronic hypertension** | -869.2 [-886.5-(-851.9)] | -853.9 [-872.5-(-853.3)] |
| **Composite event^µ^ excluding chronic hypertension** | -9.0 [-12.1-(-5.8)] | -6.7 [-10.3-(-3.1)] |
| **Chronic hypertension** | -871.0 [-888.3-(-853.7)] | -856.5 [-875.1-(-838.0)] |
| **Heart failure** | -3.5 [-5.3-(-1.8)] | -2.8 [-4.8-(-0.9)] |
| **Coronary heart disease** | -2.2 [-3.6-(-0.8)] | -1.6 [-3.2-(-0.1)] |
| **Cerebrovascular disease** | -3.8 [-6.0-(-1.5)] | -3.0 [-5.5-(-0.4)] |

*^*^ Women were considered treated if they had received at least one antihypertensive treatment in the month following maternity discharge. Women were considered non-treated if they had not received any antihypertensive treatment in the month following maternity discharge*

*^$^ Matched on the propensity score calculated based on the assessment of preterm HDP, severe HDP, and use of prenatal antihypertensive treatment*

*^§^ Difference in the mean survival time up to 10-years between treated and non-treated women, expressed in days; CI confidence interval*

*^**^ Adjusted on maternal age, multiple pregnancy, gestational diabetes, morbid obesity, severe alcoholism, personal or family history of cardiovascular disease, smoking, pre-existing diabetes and dyslipidaemia*

*^£^ Adjusted on maternal age, gestational diabetes, morbid obesity, severe alcoholism, personal or family history of cardiovascular disease, smoking, pre-existing diabetes and dyslipidaemia for composite event including chronic hypertension and for chronic hypertension; Adjusted on maternal age, gestational diabetes, morbid obesity, smoking, pre-existing diabetes and dyslipidaemia for other events because too few numbers for adjustment*

*^µ^ Defined by the occurrence of at least one cardiovascular event*

**TABLE S8.** Sensitivity analysis including only women aged 15–49: Absolute risks, traditional Cox model and extended Cox regression with a step function model (unmatched cohort).

|  |  | **Composite event^µ^ including chronic hypertension** | **Composite event^µ^ excluding chronic hypertension** | **Chronic hypertension** |
| --- | --- | --- | --- | --- |
|  |  | **n (%)** | **n (%)** | **n (%)** |
| **Number of events** | **Non-treated women N = 72 975** | 13 425 (18.40) | 794 (1.09) | 13 132 (18) |
|  | **Treated^*^ women N = 35 844** | 15 739 (43.91) | 608 (1.70) | 15 620 (43.58) |
|  |  | **aHR [95% CI]** | **aHR [95% CI]** | **aHR [95% CI]** |
| **Adjusted^**^ effects of the antihypertensive treatment at maternity discharge^*^** | **Cox model** | 3.025 [2.956-3.096] | 1.457 [1.310-1.620] | 3.066 [2.996-3.139] |
|  | **Extended Cox Step Function model** | | |  |
|  | **before 3 months** | 107.372 [87.064 - 132.418] | 3.660 [2.355 - 5.689] | 141.837 [111.468 - 180.480] |
|  | **3-6 months** | 15.555 [14.137 - 17.116] | 0.790 [0.378 - 1.651] | 16.050 [14.569 - 17.681] |
|  | **6m-1 year** | 3.316 [3.051 - 3.605] | 0.836 [0.455 - 1.535] | 3.364 [3.093 - 3.659] |
|  | **1-2 years** | 1.685 [1.554 - 1.827] | 1.448 [0.969 - 2.164] | 1.675 [1.544 - 1.817] |
|  | **2-3 years** | 1.693 [1.559 - 1.839] | 1.708 [1.142 - 2.555] | 1.699 [1.563 - 1.847] |
|  | **3-4 years** | 1.640 [1.507 - 1.785] | 1.024 [0.675 - 1.554] | 1.666 [1.529 - 1.815] |
|  | **4-5 years** | 1.401 [1.280 - 1.533] | 1.139 [0.813 - 1.597] | 1.411 [1.288 - 1.545] |
|  | **5-6 years** | 1.468 [1.335 - 1.613] | 1.674 [1.179 - 2.375] | 1.466 [1.333 - 1.613] |
|  | **6-7 years** | 1.523 [1.382 - 1.678] | 1.015 [0.715 - 1.440] | 1.549 [1.405 - 1.708] |
|  | **7-8 years** | 1.354 [1.228 - 1.493] | 1.428 [1.058 - 1.926] | 1.360 [1.232 - 1.501] |
|  | **8-9 years** | 1.427 [1.291 - 1.578] | 2.301 [1.668 - 3.174] | 1.413 [1.277 - 1.564] |
|  | **9-10 years** | 1.313 [1.190 - 1.448] | 1.338 [1.015 - 1.763] | 1.315 [1.190 - 1.451] |

*^**^ Adjusted on maternal age, multiple pregnancy, gestational diabetes, morbid obesity, severe alcoholism, personal or family history of CVD, smoking, pre-existing diabetes and dyslipidaemia*

*^*^ Women were considered treated if they had received at least one antihypertensive treatment in the month following maternity discharge. Women were considered non-treated if they had not received any antihypertensive treatment in the month following maternity discharge*

*^µ^ Defined by the occurrence of at least one cardiovascular event*

*aHR=adjusted hazard ratio; CI confidence interval*

**TABLE S9.** Sensitivity analysis including only women for whom we had access to the deprivation index: Absolute risks, traditional Cox model and extended Cox regression with a step function model (unmatched cohort).

|  |  | **Composite event^µ^ including chronic hypertension** | **Composite event^µ^ excluding chronic hypertension** | **Chronic hypertension** |
| --- | --- | --- | --- | --- |
|  |  | **n (%)** | **n (%)** | **n (%)** |
| **Number of events** | **Non-treated women N = 67 441** | 12 227 (18.13) | 733 (1.09) | 11 957 (17.73) |
|  | **Treated^*^ women**  **N = 33 315** | 14 534 (43.63) | 545 (1.64) | 14 418 (43.28) |
|  |  | **aHR [95% CI]** | **aHR [95% CI]** | **aHR [95% CI]** |
| **Adjusted^**^ effects of the antihypertensive treatment at maternity discharge^*^** | **Cox model** | 3.058 [2.985 - 3.133] | 1.405 [1.256 - 1.571] | 3.099 [3.024 - 3.175] |
|  | **Extended Cox regression with Step Function multivariate model** | | |  |
|  | **before 3 months** | 113.965 [91.240 - 142.349] | 3.048 [1.918 - 4.843] | 154.354 [119.171 - 199.924] |
|  | **3-6 months** | 16.105 [14.561 - 17.814] | 0.659 [0.279 - 1.558] | 16.550 [14.946 - 18.326] |
|  | **6m-1 year** | 3.293 [3.016 - 3.596] | 0.816 [0.425 - 1.564] | 3.335 [3.053 - 3.643] |
|  | **1-2 years** | 1.682 [1.545 - 1.830] | 1.545 [1.022 - 2.335] | 1.670 [1.533 - 1.818] |
|  | **2-3 years** | 1.682 [1.542 - 1.834] | 1.323 [0.848 - 2.066] | 1.690 [1.549 - 1.845] |
|  | **3-4 years** | 1.644 [1.504 - 1.797] | 1.086 [0.712 - 1.656] | 1.674 [1.530 - 1.832] |
|  | **4-5 years** | 1.429 [1.300 - 1.570] | 1.155 [0.809 - 1.648] | 1.435 [1.305 - 1.578] |
|  | **5-6 years** | 1.459 [1.321 - 1.611] | 1.522 [1.048 - 2.210] | 1.461 [1.322 - 1.615] |
|  | **6-7 years** | 1.479 [1.336 - 1.637] | 0.913 [0.634 - 1.314] | 1.511 [1.364 - 1.674] |
|  | **7-8 years** | 1.350 [1.219 - 1.497] | 1.503 [1.098 - 2.058] | 1.340 [1.208 - 1.487] |
|  | **8-9 years** | 1.431 [1.288 - 1.590] | 2.303 [1.658 - 3.198] | 1.411 [1.269 - 1.570] |
|  | **9-10 years** | 1.286 [1.160 - 1.426] | 1.317 [0.988 - 1.755] | 1.286 [1.158 - 1.427] |

*^**^ Adjusted on maternal age, multiple pregnancy, gestational diabetes, morbid obesity, severe alcoholism, personal or family history of CVD, smoking, pre-existing diabetes*

*dyslipidaemia, and deprivation index*

*^*^ Women were considered treated if they had received at least one antihypertensive treatment in the month following maternity discharge. Women were considered non-treated if they had not received any antihypertensive treatment in the month following maternity discharge*

*^µ^ Defined by the occurrence of at least one cardiovascular event*

*aHR=adjusted hazard ratio; CI confidence interval*

**TABLE S10.** Sensitivity analysis excluding women with preterm or severe hypertensive disorders of pregnancy: Absolute risks, traditional Cox model and extended Cox regression with a step function model (unmatched cohort).

|  |  | **Composite event^µ^ including chronic hypertension** | **Composite event^µ^ excluding chronic hypertension** | **Chronic hypertension** |
| --- | --- | --- | --- | --- |
|  |  | **n (%)** | **n (%)** | **n (%)** |
| **Number of events** | **Non-treated women N = 55 438** | 10 596 (19.11) | 562 (1.01) | 10 399 (18.76) |
|  | **Treated^*^ women N = 20 602** | 9 294 (45.11) | 330 (1.60) | 9 232 (44.81) |
|  |  | **aHR [95% CI]** | **aHR [95% CI]** | **aHR [95% CI]** |
| **Adjusted^**^ effects of the antihypertensive treatment at maternity discharge^*^** | **Cox model** | 2.981 [2.899-3.066] | 1.452 [1.266-1.665] | 3.015 [2.931-3.101] |
|  | **Extended Cox regression with Step Function multivariate model** | | |  |
|  | **before 3 months** | 116.969 [90.204 - 151.677] | 3.577 [2.078 - 6.157] | 161.473 [119.041 - 219.030] |
|  | **3-6 months** | 16.089 [14.333 - 18.060] | 0.724 [0.267 - 1.963] | 16.650 [14.809 - 18.719] |
|  | **6m-1 year** | 3.432 [3.099 - 3.800] | 1.759 [0.781 - 3.961] | 3.442 [3.107 - 3.812] |
|  | **1-2 years** | 1.690 [1.530 - 1.868] | 1.581 [0.957 - 2.613] | 1.688 [1.527 - 1.867] |
|  | **2-3 years** | 1.767 [1.601 - 1.952] | 1.577 [0.934 - 2.662] | 1.770 [1.602 - 1.956] |
|  | **3-4 years** | 1.723 [1.558 - 1.906] | 1.083 [0.622 - 1.886] | 1.742 [1.574 - 1.927] |
|  | **4-5 years** | 1.488 [1.336 - 1.657] | 1.384 [0.925 - 2.071] | 1.494 [1.341 - 1.666] |
|  | **5-6 years** | 1.608 [1.436 - 1.801] | 1.505 [0.941 - 2.408] | 1.626 [1.451 - 1.822] |
|  | **6-7 years** | 1.613 [1.435 - 1.812] | 1.012 [0.646 - 1.584] | 1.625 [1.445 - 1.829] |
|  | **7-8 years** | 1.359 [1.205 - 1.534] | 1.438 [0.987 - 2.094] | 1.366 [1.209 - 1.542] |
|  | **8-9 years** | 1.482 [1.311 - 1.675] | 1.882 [1.254 - 2.826] | 1.463 [1.292 - 1.656] |
|  | **9-10 years** | 1.299 [1.151 - 1.466] | 1.170 [0.807 - 1.696] | 1.309 [1.159 - 1.478] |

*^**^ Adjusted on maternal age, multiple pregnancy, gestational diabetes, morbid obesity, severe alcoholism, personal or family history of CVD, smoking, pre-existing diabetes and dyslipidaemia*

*^*^ Women were considered treated if they had received at least one antihypertensive treatment in the month following maternity discharge. Women were considered non-treated if they had not received any antihypertensive treatment in the month following maternity discharge*

*^µ^ Defined by the occurrence of at least one cardiovascular event*

*aHR=adjusted hazard ratio; CI confidence interval*
